# The Efficient Measurement of Job Satisfaction: Facet-Items versus Facet Scales

**DOI:** 10.3390/ijerph15071362

**Published:** 2018-06-28

**Authors:** Angelika Lepold, Norbert Tanzer, Anita Bregenzer, Paulino Jiménez

**Affiliations:** Department of Psychology, University of Graz, 8010 Graz, Austria; angelika.lepold@edu.uni-graz.at (A.L.); norbert.tanzer@uni-graz.at (N.T.); anita.bregenzer@uni-graz.at (A.B.)

**Keywords:** workplace health promotion, job satisfaction, facet-items, facet scales, measurement, effectiveness

## Abstract

The measurement of job satisfaction as a central dimension for workplace health and well-being is crucial to set suitable health- and performance-enhancing management decisions. Measuring different facets of job satisfaction leads to a more precise understanding about job satisfaction in research as well as to more specific interventions in companies. This study examines the measurement of job satisfaction with facet scales (multiple-items for one facet) and facet-items (one item for one facet). Facet-items are a cost-effective and fast way to measure job satisfaction in facets, whereas facet scales are more detailed and provide further information. Results from 788 bank employees showed that facet-items of job satisfaction were significantly correlated with the corresponding facet scales and had high factor loadings within the appropriate factor. Furthermore, the same correlational pattern between facet scales and external criteria was found for facet-items and external criteria (identification with the company, work engagement, stress, resources). The findings support the usage of facet-items in companies and in research where cost- and time-effectiveness is imperative and the usage of facet scales where an even deeper understanding of job satisfaction is needed. In practice, the usage of efficient measurements is evident, especially in the upcoming field of eHealth tools.

## 1. Introduction

Is your job making you feel satisfied? Job satisfaction (JS) is one of the most studied fields of work design research in psychology [1]. The wide interest of JS is valid for research and for organizations [2]. JS can be seen in two different ways: On one hand, JS is used as a measurement for well-being of employees [3]. In this respect, JS displays the emotional state of the employees that is also frequently used as an indicator in workplace health promotion projects to develop specific interventions [4]. On the other hand, JS is seen in a dynamic process as a predictor and outcome variable for other job-related factors in the direction of performance [5,6], e.g., work engagement. Since engaged employees are more productive [7], organizations want to explore the level of JS of their employees. High JS can be a goal for organizations to reduce their fluctuations and to enhance their performance, but for the employees by themselves, it is of deep interest to enhance their own quality of life. JS is defined as a pleasurable or positive emotional state [8] and is developed through evaluative judgments, affective experiences at work, and beliefs about jobs [9]. It is associated with important work-related and general outcomes [10,11] as e.g., JS shows a high variance proportion for the prediction of general life satisfaction [12]. Furthermore, JS and work engagement show a high positive relationship [13]. Therefore, the measurement of JS plays a fundamental role as organizations want highly involved and satisfied employees to reach their goals (e.g., [14]). The measurement of JS gives insight into the attitudes of the employees in the company and can be used to support the design of corporate strategies [15]. 

A typical way of using JS is by including it as organizational diagnostic variable in employee surveys [16] to derive actions according to the goals and strategies of the organization. These goals can be derived with the Balanced Scorecard [17]. The Balanced Scorecard is an instrument to translate the strategies and goals of a company into precise measurable indicators. The learning and growth perspective of the Balanced Scorecard, which is one of four perspectives, is the foundation for all other perspectives. Investments in further education, information technologies, and systems are part of the learning and growth perspective where the base to reach other defined goals is set. The learning and growth perspective includes, amongst other indicators, the satisfaction of the employees to increase productivity and quality [18], where JS can be measured with an employee survey [19]. Recent research shows that economic measurement is important to get information and to decrease the reaction time to set actions in a company [20]. 

As part of most employee surveys, JS can be on one hand considered as a global construct or on the other hand can be seen as consisting of various facets [21], where, according to Judge and Kammeyer-Mueller [22], JS is a hierarchical construct. Global JS combines all feelings and cognitions toward the job whereas the facet approach considers various aspects of the job like work task, payment, promotion, supervision, or coworkers [15,21]. These different approaches lead to various measurements of JS [23]. We differentiate the approaches into the goal of assessing global JS or facets of JS as presented in Table 1.

The contrasts between these approaches can be seen at different levels. JS is often measured with multiple-items aggregated to global JS [24]. A new way of assessing JS is with one single-item asking for global JS (e.g., [25]). Global JS is an efficient way of measuring well-being either in a single-item version or with multiple-items, where the advantage of multiple-items lies in higher reliability [26]. Measuring dimensions (or facets) of JS can be done with multiple-items aggregated to different facets of JS [27,28]. Assessing facets of JS is useful to get a deeper insight into the different organizational processes [14] as also the multidimensional complexity of JS has to be taken into account [29,30]. The facets that are considered, e.g., by the Job Descriptive Index [27], are the work by itself, compensation and benefits, attitudes toward supervisors, relations with co-workers, and opportunities for promotion. But there can be other facets like working conditions [8]. Given the current measurements of JS, there exist different facets of JS as well as different compositions of facets [15]. Regarding the idea of an efficient way of measuring JS, which at the same time covers all important facets, can be seen best presented with the use of single-items where every item describes one facet of JS, e.g., the facet “satisfaction with working conditions” is assessed with one item (e.g., [20,31]). We define single-items, which are used as assessment for a single facet of JS, as facet-items.

The aim of this study is to introduce and test a set of JS instruments: an instrument of JS consisting of different facet scales (Profile Analysis of Job Satisfaction (PAJS); [28]) and a specially developed screening version are compared. This screening version of the measurement of JS consists of a battery of facet-items, where every item represents one facet of the facet scales. Instruments with facet-items have been developed before (e.g., [20,31]) but have included only a small range of possible facets, focusing strongly on facets measuring JS with social aspects (supervisor, coworkers) or with task-related aspects (work tasks, payment, career possibilities). To get a deeper insight into the workplace, a more detailed assessment is needed where a wide range of possible facets must be considered (e.g., the information processes, the working and vacation times, or working conditions). The PAJS contains 11 facets with multiple-items for every facet and, therefore, allows a very specific assessment of the workplace. A screening version with one item for each of the 11 facets could provide more information than single- or multiple-items of global JS and be at the same time more cost- and time-efficient than the PAJS. Nonetheless the usage of multiple-item measurements for JS is necessary to get a deeper insight into the facets and to derive interventions more precisely.

### 1.1. Measuring Job Satisfaction with Facet-Items versus Facet Scales

Regarding single-items of JS, they need less time and space, are more cost-effective, and may contain more face validity than scales with multiple-items [32]. Cost- and time-effectiveness is an important issue for companies. Shorter surveys are more likely to be approved by companies and are more likely to be completed by the employees or participants in a study [33,34]. Therefore, an economic measurement will lead to higher participation of employees in an employee survey or in research studies [20]. These advantages can also be assumed for facet-items of JS [31]. 

Criticism against single-item measurements refer to the assessment of reliability: the test-retest-reliability for single-item measurements can be estimated, but the more psychometrically important estimation of internal consistency cannot be generated [26]. Furthermore, Wanous et al. [32] report in their meta-analysis test-retest-reliabilities for single-items between 0.45 and 0.69, which is a large bandwidth. But as Gardner et al. [35] state, it is possible that one “good” item shows better reliability and validity than many “bad” items. Wanous et al. [32] report correlations at 0.63 between single-items and scale measures showing that single-items of JS have an acceptable psychometric quality. Besides that, not only reliability but also validity for the practical usage of scales is at least of high importance [36].

For other constructs, like work engagement, it was already shown that a short and efficient measurement can be used [13]: the Utrecht Work Engagement Scale (UWES) measures the three dimensions of vigor, dedication, and absorption with one item for each dimension and shows acceptable reliability and validity. For narcissism, a single-item measurement was also developed, showing high test-retest-reliability and acceptable validity [37]. Even assessing the health status with global single-items as a valid, reliable, and sensitive measurement can be done [38]. 

In the present study, facet-items of JS are direct statements. Facet-items in form of questions in a discrepancy approach [31] can lead to psychometric problems [39]. Furthermore, the used measurement, in fact the PAJS, contains 11 facets of JS, and therefore, it is even more comprehensive and easier to derive practical interventions than with less facets. As the measurement of 11 facets with multiple-items for every scale is costly, a facet-item approach is more efficiently and less cost-intensive. Therefore, a screening version was developed to measure the facets of JS with facet-items: 11 items were defined to measure 11 facets. The items included the name of the facet scale and in parentheses further descriptions of the scale (this is presented in Table 2, see third column). To be efficient, the circumstance of having items measuring more than one aspect was deliberately accepted, but for that more comprehensible items were possible. 

### 1.2. Job Satisfaction and External Criteria

On one hand, JS is used as an indicator for well-being, e.g., as an output of changes within a company inducing organizational health performance interventions [40]. On the other hand, JS is supposed to be a cause for other outcomes like absenteeism, performance, productivity, work engagement, organizational inefficiency (like counterproductive behaviour), intention to quit, or commitment [11,21,41]. In the applied field of organizational psychology, JS is used as a broad indicator of these outcomes or as an indicator of well-being, concluding that any measurement of JS either as facet-items or facet scales has to be tested with the respective outcomes as external criteria. The facets of JS lead to different predictions of behaviour and therefore, different interventions can be derived [20]. In an international study for example the relational aspect of JS has been identified as the most important facet for performance [42] and in a study among senior managers in the forestry and wood-processing sector, base salary was the most important factor for motivation [43]. By using organizational-specific results like the previous one then subsequent steps for changes in an organization can be developed in a tailored manner.

Organizational identification, declared as a strong affective and cognitive bond between employee and organization, shows a positive relationship with JS [44], whereas intention to quit is related negatively to JS [41]. The positive relationship between organizational identification and JS also occurs when JS is summarized from different facets [45]. Jiménez [46] states that different facets of JS have influence on intention to quit and organizational identification. Satisfaction with making career and satisfaction with how demanding the job is are the most important influence factors for organizational identification. 

Furthermore, JS and work engagement can be viewed as two different constructs in organizational psychology with a positive relationship [13,47] but higher levels of arousal for work engagement than for JS [13]. The importance of work engagement for todays’ work environment has been proved in many studies [13]. Different facets of JS are meant to make different predictions of work engagement, e.g., satisfaction with the work by itself is the key driver of work engagement, whereas satisfaction with payment is not linked to work engagement [48]. 

Projects in workplace health promotion aim to enhance resources and to reduce stress. In the end, these projects should improve well-being, often measured in the form of JS [49]. As job resources are a positive feature of work environment related to motivational outcomes, resources are of an outstanding interest for companies [50,51] and are further related to well-being [52]. Stress, seen as a process caused by a load beyond the level of normal functioning [53], is an indispensable part in workplace health promotion projects. It has been shown that stress and JS are negatively related, whereas the different facets of JS are differently important [54].

### 1.3. Research Questions

#### 1.3.1. Facet-Items in Comparison with Facet Scales of Job Satisfaction

One aim of this study is to compare a facet-item measurement with a facet scale measurement of JS. The used facet scale measurement, the PAJS, is a standardized approach to measure a wide range of facets of JS and it is possible to compare the results of an organization with a representative sample. It is hypothesized that facet-items are significantly correlated to the appropriate facet scale of the PAJS. Furthermore, intercorrelations between the facets of JS should be moderate [55]. In addition, the facet-items should show high factor loadings within the appropriate factor in a confirmatory factor analysis with all items of the PAJS. The findings should provide evidence to use a facet-item approach to measure JS where cost- and time-effectiveness is imperative.

#### 1.3.2. Facets of Job Satisfaction and External Criteria

In addition, as the validity of a measurement must be checked, this is another aim of the present study. To test validity, the facet-item measurement as well as the facet scale measurement is related to other external criteria. External criteria in this study are identification with the company, work engagement, stress, and resources. Correlations between external criteria and the two measurements of JS should show similar patterns. To interpret the correlation coefficients, the classification from Cohen [56] was used, pointing out correlations higher than 0.30 are moderate and correlations higher than 0.50 are high. The level of correlations between JS and organizational identification should be moderate [44]. Furthermore, relationships between JS and work engagement should be moderate to high [13,47] as well as relationships between JS and resources and stress [54]. 

#### 1.3.3. Efficiency of a Facet-Items Approach

To prove the efficiency of the newly developed facet-items approach compared to a facet scales approach, the comparison between the average answer times of the two measurements is another aim of the present study. It is hypothesized, that the average answer time for the facet-items measurement is shorter than for the facet scales approach. 

## 2. Materials and Methods

### 2.1. Participants and Procedure

The participants in this online-study were 788 employees working for an Austrian bank. The overall response rate was 53% (total of 1495 employees). The study is a first result in a longitudinal project about workplace health promotion (“Employee Survey 2015—Main Focus on Psychosocial Risk Management according to the Austrian Employee Protection Act”) and was conducted in the year 2015. The participation in the workplace health promotion project was voluntary. Nearly half of the employees were female (49.4%), and the others were male (50.6%). The largest portion had no leading position (74.6%). In this study, 13.8% of the bank employees had no contact to clients (employees in the back office), and 74.2% worked full-time, the others part-time. Age was measured in four categories: 13.1% were between 21 and 30 years old, 22.7% between 31 and 40 years, 33% between 41 and 50 years, and 31.2% were older than 50 years.

The survey was advertised on the intranet of the bank and through e-mail. The bank employees were asked to participate in the workplace health promotion project (including an employee survey) that takes place every two years and is conducted by an external, independent research institute. The first part of the survey included the questionnaires of the typical part of the employee survey, and in the second part, employees were asked to participate in a research project from the University of Graz to get some information about the effects of JS. At first, participants had to rate their JS measured with a screening version (measurement of facet-items). The second part of the survey included the PAJS (measurement of facet scales). The participation in the study was completely voluntary, anonymous, and confidential. Participants were promised total data protection. The study was carried out in accordance with the recommendations of the guidelines of the Ethics Commission of the University of Graz and approved by the Ethics Commission of the University of Graz from 27 March 2015.

### 2.2. Measures

#### 2.2.1. Job Satisfaction (Facet Scales and Facet-Items)

The PAJS by Jiménez [28] with 38 items belonging to 11 facets was used to measure the facets of JS with several items for every facet (facet scales). Three to five items belong to every facet of the PAJS. This scale for job satisfaction (published at a test publisher [28]) has been used in organizational diagnostic studies in research and in practice. Studies showed Cronbach’s alpha for the facets of JS measured with the PAJS from 0.82 to 0.91 [28,57]. Criterion validity for the PAJS showed in different studies a negative relationship of JS with burnout, intention to quit, or absenteeism [28,58]. Furthermore, construct validity was proven with the Job Diagnostic Survey by Hackman and Oldham [59,60]. The practical requirements often requested for a shorter version with high psychometric quality.

Based on the ideas of being efficient (cost and time), a screening version was developed with the items of the PAJS to measure the facets of JS with facet-items. Therefore, it was accepted to have items measuring more than one aspect, but for that getting more comprehensible items. With the idea of being efficient, for the PAJS-Facet-Item (PAJS-FI) eleven items were developed to measure eleven facets. The development of the facet-items included the name of the facet scale and in parentheses the single-items of the facet scales or other descriptions of the scale. As an example, the facet *information and communication* with three single-items of the PAJS was measured in the PAJS-FI with the facet-item “I am … (rated from “1, very satisfied” to “5, unsatisfied”) with information and communication (activities in company, treatment of my suggestions, information from the management, information about innovations)”. Another example for the facet organization and management is the facet-item “I am … (rated from “1, very satisfied” to “5, unsatisfied”) with the organization and management (effort regarding employees, participation possibilities, image)”. It seems on one hand to be trivial to compare these two measurements, on the other hand it is important for practice and economic science to get valuable results for the practical use of efficient measurements in employee surveys with scientific accuracy [61]. In Table 2, the different facets as well as the facet-items from the PAJS-FI and sample items from the facet scales of the PAJS are shown. The facet to which the items belong is also represented in Table 2. 

Employees were asked to indicate their agreement on a five-point scale (1 = very satisfied, 5 = unsatisfied). For an easier interpretation of the results, the values were inverted to get high values referring to high JS. In the present study, the Cronbach’s alpha for the global JS score in PAJS-FI was α = 0.89 and for the PAJS also α = 0.89.

#### 2.2.2. Identification with the Company

Identification with the company was measured with four different items [28,47] which were adapted to the employee survey in the company and contained the aspects of recommendation and reapplying to the company, identification with the company, and proudness of working in this company. The items had to be rated on a five-point scale (1 = does apply perfectly, 5 = does not apply at all). For an easier interpretation, the values of identification with the company were inverted to get high values referring to high identification. A sample item is “I identify myself strongly with the company”. Cronbach’s alpha for identification with the company was α = 0.90. 

#### 2.2.3. Work Engagement

Work engagement was measured with the short version of the UWES [62]. The UWES-9 includes nine items pertaining to three dimensions: *vigor*, *dedication*, and *absorption* (three items for each dimension). Participants were asked to rank their answers on a seven-point scale from 0 (never) to 6 (always/every day). Sample items are “At my work, I feel bursting with energy” (vigor), “I am proud on the work that I do” (dedication), and “I feel happy when I am working intensely” (absorption). Cronbach’s alpha for work engagement was α = 0.96.

#### 2.2.4. Resources and Stress

Resources and stress were measured with the Recovery-Stress-Questionnaire for Work [63]. With this measurement, the two dimensions stress and resources can be displayed. In the present study, a short version of the RESTQ-Work (RESTQ-Work-27) was used. For the dimension stress, the sub-dimensions social emotional stress and loss of meaning can be generated. The dimension resources contains the sub-dimensions overall recovery, leisure/breaks, psychosocial resources, and work-related resources. Employees were asked to rate their answers from 0 (never) to 6 (always). A sample item for the dimension resources is “In the last 7 days and nights I felt physically relaxed” and a sample item for the dimension stress is “In the last 7 days and nights I felt down”. Cronbach’s alpha for resources was α = 0.93 and for stress α = 0.94.

## 3. Results

Data were analysed using Version 24 of Statistical Package for Social Sciences (SPSS (IBM SPSS software, Armonk, NY, USA) and using the program Mplus (Version 7.3 (Muthén & Muthén, Los Angeles, CA, USA)). 

### 3.1. Relationship between Facets of PAJS (Facet Scales) and PAJS-FI (Facet-Items)

To test if facet-item measures belong to the appropriate facet scale, Pearson correlations between the PAJS-FI facet-items and the PAJS facet scales (scales obtained by aggregation of single-items) were calculated in SPSS. In Table 3, the correlations between the facets of the PAJS and the PAJS-FI are displayed. 

Every facet-item showed a moderate to high correlation with the appropriate facet scale, ranging from 0.50 to 0.82 (*p* < 0.01). The lowest correlation between facet-items and facet scales was shown for the facet general framework conditions (0.50, *p* < 0.01) and the highest correlation for the facet relationship to direct supervisor (0.82, *p* < 0.01). By further examination of the correlations, it can be seen that the facet-item organization and management correlates higher with the facet scale relationship to direct supervisor (0.67, *p* < 0.01) than with the facet scale organization and management (0.54, *p* < 0.01). 

The intercorrelations between the facets of the PAJS as well as between the PAJS-FI were small to high and ranged from 0.20 to 0.70 (*p* < 0.01) for PAJS-FI and from 0.24 to 0.64 (*p* < 0.01) for PAJS.

### 3.2. Confirmatory Factor Analysis for PAJS (Facet Scales) and PAJS-FI (Facet-Items)

To test if the facet-items of the PAJS-FI belong to the same factor as the single-items of the facet scales of the PAJS and show high factor loadings, a confirmatory factor analysis was conducted. For every facet of JS, the single-items of the PAJS and the facet-item of the PAJS-FI belonging to the same facet, were modelled as one factor. All 11 facets were put into one higher-order factor. The confirmatory factor analysis was conducted in the program Mplus.

In Table 4, the standardized factor loadings of PAJS-FI facet-items with the appropriate facet are shown. The factor loadings of the single-items of the facet scales (PAJS) are not shown in Table 4. Standardized loadings for the items of the PAJS-FI reached from 0.64 for the facets general framework conditions and organization and management to 0.85 for the facets relationship to direct supervisor and relationship to direct colleagues. Model fit also showed appropriate results: χ^2^ (1116) = 4300.86, CFI = 0.90, SRMR = 0.07, RMSEA = 0.06.

### 3.3. The Facets of Job Satisfaction and External Criteria

To test validity, the facet-items of the PAJS-FI were correlated with external criteria. The correlations of the external criteria with the facet-items of PAJS-FI were compared to the same correlations between the facet scales of the PAJS and the same external criteria. As external criteria identification with the company, work engagement, stress, and resources were used. In Table 5, the Pearson correlations between the facet-items of the PAJS-FI and the facet scales of the PAJS with the external criteria identification with the company, work engagement, stress, and resources, calculated in SPSS, can be found. To test if differences between the correlations from PAJS and PAJS-FI with the respective external criteria exist, a test of the difference between two dependent correlations with one variable in common was calculated [61,64,65]. For that reason, Bonferroni correction was applied [66,67], and the results were compared with α = 0.0004 (0.05 divided by 143 possibilities). Results showed significant differences for several facets especially for organization and management (see Table 5).

The correlations between the different items of identification with the company and the facet-items of the PAJS-FI ranged from 0.23 (relationship to direct colleagues and identify with the company) to 0.44 (demanding work and reapply at the company, chances of making career and proud to work at the company). Correlations between the facet scales of the PAJS and the items of identification with the company reached from 0.27 (relationship to direct colleagues and identify with the company) to 0.65 (organization and management and recommendation of the company). The correlations between identification with the company and the facet scales (PAJS) were always slightly higher than with the facet-items of the PAJS-FI except for a few single correlations (mainly for the facet compensations of the employer). The pattern of the correlational structure remained the same for PAJS and PAJS-FI. Significant results between PAJS and PAJS-FI were found for the facet organization and management. These differences were analysed additionally and are described below. 

For work engagement, the correlations for the facet-items of the PAJS-FI ranged from 0.23 (relationship to direct colleagues and absorption) to 0.61 (demanding work and dedication). The facet scales of the PAJS and work engagement showed correlations from 0.26 (compensations of the employer and absorption) to 0.66 (demanding work and dedication). The aforementioned correlations were slightly higher than with the facet-items of the PAJS-FI except for the facet compensations of the employer, but the correlational structure was the same for both measurements. Significant results were found for the facets information and communication, organization and management, chances of making career, working and vacation times, and general framework conditions. 

Concerning stress (sub-dimensions social emotional stress and loss of meaning) the correlations between the facet-items of the PAJS-FI and stress reached from −0.30 (compensations of the employer and social emotional stress) to −0.50 (organization and management and loss of meaning). For the facet scales of the PAJS and stress the correlations reached from −0.21 (compensations of the employer and social emotional stress) to −0.60 (decision range and loss of meaning), higher than the correlations with the facet-items of the PAJS-FI except for the facets organization and management, chances of making career, working conditions, compensations of the employer, and general framework conditions. Significant differences via Steiger’s Equations were found for the facets decision range as well as compensations of the employer.

Resources (sub-dimensions overall recovery, leisure/breaks, psychosocial resources, work-related resources) showed correlations from 0.20 (demanding work and psychosocial resources) to 0.68 (relationship to direct colleagues and psychosocial resources) for the facet-items of the PAJS-FI. For the facet scales of the PAJS, the correlations ranged from 0.18 (compensations of the employer and psychosocial resources) to 0.72 (relationship to direct colleagues and psychosocial resources). Most of the facets showed higher correlations for the facet scales of the PAJS and resources than for the facet-items of the PAJS-FI except for a few single correlations. The structural pattern for the facet scales (PAJS) and the facet-items (PAJS-FI) with stress and resources was the same. Significant differences were found for the facets relationship to direct colleagues as well as compensations of the employer.

In sum, the correlational structures between the facet scales (PAJS) and external criteria were the same like between facet-items (PAJS-FI) and external criteria. Most correlations showed a moderate to high level, except for the facets compensations of the employer, working and vacation times, and relationship to direct colleagues, and a few single correlations.

As the most significant comparisons between PAJS and PAJS-FI resulted for the facet organization and management, further reliability analyses were explored. The internal consistency for the three single-items for the facet organization and management from PAJS was α = 0.85, whereas reliability analysis including the facet-item organization and management showed α = 0.83 (analysed with Cronbach’s alpha). A more stricter analysis with McDonald’s coefficient omega (omega hierarchical; [68]) showed ωh = 0.85 vs. ωh = 0.78. Furthermore, item selectivity was lowest for the facet-item. These coefficients present that the reliability is stronger in the multiple-item version compared to the reliability including the facet-item.

### 3.4. Efficiency of the Short Version PAJS-FI

A *t*-test for dependent samples was calculated to see if the answer time for the measurement PAJS is different from the answer time for the PAJS-FI. People who interrupted the survey were excluded from the analysis. The test showed that for answering the items of the PAJS (*M* = 195.92 s, *SD* = 188.24), the answer time lasted significantly longer than for the facet-items of the PAJS-FI (*M* = 43.74 s, *SD* = 83.52, *t*(577) = −18.34, *p* < 0.01). The answer time was measured in seconds.

## 4. Discussion

This paper aimed to test a comprehensive facet-item measurement of JS that includes the assessment of eleven facets of JS. Furthermore, the facet-item measurement was compared to a facet scale measurement of JS to analyse construct and criterion validity. The results showed that the facet-items of JS were significantly and highly related to the facet scales. Additionally, results of a confirmatory factor analysis showed that the facet-items loaded highly within the appropriate factor for each facet scale. Furthermore, correlations with external criteria were acceptable showing valid measurements. 

### 4.1. Comparison between Facet-Items and Facet Scales of Job Satisfaction

JS has been introduced as a global construct measured with one single-item or with multiple items or as a construct representing different facets measured with facet-items or with multiple items aggregated to facets (facet scales). The usage of facets is useful for a deeper understanding of organizational processes [14], and therefore, efficiency plays a major role. 

Regarding the first research question, we conclude with the high correlations of the facet scales with the facet-items (diagonal in bold in Table 3) that both versions can be used for their different usages. This seems at first sight trivial, but the goal of the study was to develop an efficient measurement for practical use without forgetting precise, scientific standards. Therefore, the simple correlations were analysed, and additionally, a confirmatory factor analysis has been conducted. As expected, the correlations between the facet scales of PAJS and the facet-items of the PAJS-FI were high, and it is summarized that the PAJS-FI showed appropriate correlations for the eleven facets. Moreover, confirmatory factor analysis showed for all eleven facets that the facet-items of the PAJS-FI had high loadings within the appropriate factor. Single-item approaches, like the PAJS-FI for facets of JS, are useful where cost- and time-effectiveness plays an important role [20], whereas multiple-item measurements, like the PAJS, are needed in research and organizations to measure relatively complex constructs reliably [26]. 

Looking into the details, it can be seen, that the facet-item organization and management showed a high correlation with the facet scale relationship to direct supervisor (0.67). This is not surprising as the organization and the management of a company has much in common with the supervisors of a company, because the management depends on supervisors. Moreover, employees can assess their direct formal leaders better than management [69]. On the contrary, the facet scale organization and management did correlate at a moderate level (0.37) with the facet-item relationship to direct supervisor. Closer examined, the facet-item organization and management includes the nearer description “efforts regarding employees” (see Table 2). Such efforts depend on people who manage a company. The employees possibly have their supervisor in mind when thinking about management and do not include the satisfaction with the organization in their assessment. We suggest to strengthen the aspect of satisfaction with the organization in a next version of the PAJS-FI. 

### 4.2. Job Satisfaction and External Criteria

By looking at the correlations with external criteria (identification with company, work engagement, stress and resources) to answer the second research question, it was shown that the correlations visible looked higher for the facet scale approach, but not in a statistical comparison of the different correlations. The level of correlations was mostly moderate to high, except for the facets compensations of the employer, working and vacation times, and relationship to direct colleagues, and a few single correlations. The correlations showed that the level of correlations for different facets of JS with external criteria is different, as hypothesized. 

The correlational structure remained the same for the facet scales of the PAJS and the facet-items of the PAJS-FI. The higher identification with the company was, the higher was JS, no matter which measurement (PAJS or PAJS-FI) was used. For identification with the company, the facet organization and management showed significantly higher correlations with PAJS than with PAJS-FI. As already suggested, the facet organization and management in PAJS-FI should be further investigated as Cronbach’s alpha dropped when including the facet-item. Work engagement seems to have much in common with JS [48]. Here, also the correlational structure for PAJS and PAJS-FI remained the same: the higher work engagement was, the higher was JS. Stress (sub-dimensions social emotional stress and loss of meaning) and JS shared a negative relationship for the facet scales of the PAJS as well as for the facet-items of the PAJS-FI, whereas the relationship between resources (sub-dimensions overall recovery, leisure/breaks, psychosocial resources, work-related resources) and JS was positive. Summarized, the PAJS-FI and PAJS showed similar internal consistencies whereas the PAJS-FI has a shorter test-length. 

The correlations with external criteria were the same for the facet-items of the PAJS-FI and for the facet scales of the PAJS, except for a few correlations and especially for the facet organization and management. Schaufeli et al. [13] also concluded in their approach of getting a shorter version of the UWES scale, that an expectable consequence and drawback of this shortening is that the coefficient alpha is reduced. We therefore made a deeper analysis for the facet organization and management and found a lower internal consistency in this facet which may explain the differences between the two versions of the PAJS. A facet scales approach is more accurate and therefore, different correlations may remain between the two compared measurements. On the other hand, the correlational structure is in the same direction as described before. The differences have to be kept in mind when interpreting the results with the PAJS-FI. 

### 4.3. Efficiency of the PAJS-FI

As it was shown, the relative answer time for PAJS was much longer than for PAJS-FI. To answer the PAJS-FI, employees needed 44 s whereas the answer time for the items of the PAJS took more than three minutes. An efficient measurement should not be to simply have a shorter version replacing a longer version with the drawback of possibly losing information. Instead, the shorter version should lead to approximately the same information and show first results, e.g., in employee surveys. Shorter measurements meet the demands of survey participants as otherwise research has to assess fewer constructs or assess constructs with fewer items [13]. In employee surveys it is useful to have short measurements to gain as much information as possible [32,61] as there are often also time constraints [61]. On the other side, the more precise the facets of JS are measured, the more detailed interventions in a company can be derived. Therefore, the usage of multiple-item measurements of JS is as legitimate as the usage of facet-items for efficient measurement. 

### 4.4. Limitations

One limitation in this study is the specific sample of employees working in the bank sector. In agreement with the participating bank, there is no permission to show descriptive data (means and standard deviations), only the relative relationships. As the interest of this study was to compare the two different versions PAJS and PAJS-FI, and not to show specific results from bank employees, the relative relationships suffice. For this study, it was important to test a sample that can participate in a study where the PAJS and the PAJS-FI are presented at one measure point. This leads to another limitation in the study: the completion of PAJS and PAJS-FI was not randomized. Bank employees had to first fill out the PAJS-FI, and in a second step, they had to complete the PAJS. 

External criteria like job performance or turnover-rates and their relationship with facet-items of JS might be interesting and should be further investigated. Moreover, test-retest reliabilities would be of further interest. 

### 4.5. Practical Implications: Advantages and Disadvantages—The Right Placement for the Right Instrument

JS is seen as a predictor for various outcomes [11] and is therefore a useful variable for organizational development and hence supports making management decisions [2]. But also as an indicator for well-being, JS is important [57]. This can be seen in dynamic views of JS e.g., from Büssing and Bissels [5] or Jiménez [6]. These system theoretical oriented views consider especially the facets of JS and draw the attention to the qualitative forms of JS. A result of JS can be seen in a “satisfied” rating but this judgment possibly could be evaluated as a “resigned work satisfaction” [5]. Knowing more about different aspects of the working life with facets of job satisfaction supports to understand possible coping strategies of employees which in turn influence the organization. 

Employee surveys, especially when measuring JS, are an important instrument for organizational diagnosis by using the subjective assessment of a company [11]. Such employee surveys should be adopted to companies and therefore, the results should lead to topics discussed in the management area of the company and to implementations in the strategy of a company, e.g., in the Balanced Scorecard [16]. 

There are many advantages for a facet-item approach to measure the facets of JS: Cost-effective and short questionnaires need less time and space [32], and the response rate may be higher for shorter questionnaires [33]. According to Schaufeli et al. [13], shorter forms of questionnaires help to fulfil the requirements of employee surveys in companies: Due to time constraints, researchers either need to assess fewer constructs or have to assess the constructs with fewer items, which is especially the case in employee surveys. In this case, the usage of PAJS-FI is supported. Of course, multiple-item measures of the facets of JS (facet scales) have their use and advantages. They are because of the single-items even more differentiated for the employees as well as for the companies to generate interventions. One fundamental advantage of multiple-item measures is the estimation of internal consistency reliability, where high reliability is essential for statistical analysis to minimize effects of error [26]. In cases where small differentiations and the estimation of reliability is needed, the usage of PAJS is advised. 

Another application area for efficient and short measurements is in the upcoming field of eHealth tools. In this area shorter “scales” and even visual analogue scales are also used as single-item measures [70] and can be part of workplace health promotion projects. With eHealth tools using visual analogue scales or the approach of facet-items, it is possible for supervisors to get short and efficient feedback [71]. This feedback has to be valid and accurate despite usage of short versions [72]. 

## 5. Conclusions

Facet-item and facet scale measurements of JS have both their areas of application. The aim of this study was to show that a facet-item measurement can be a replacement for a facet scale measurement of JS with multiple-items. In an online study, bank employees filled out both versions of assessments of JS, and by comparing the structural resemblance of the two measurements, it was demonstrated that facet-items are an appropriate approach. Facet-items can be an approach to measure the facets of JS in employee surveys efficiently, but facet scales are also appropriate when the facets should be measured more accurately. Which approach is used depends on the goals that should be reached. In projects where efficiency plays a major role, facet-item measures are preferred. This is especially the case in larger studies [32] or in practice when using eHealth tools [70]. In research or organizations where an even deeper understanding of JS with the aim of deriving interventions is needed, facet scale measures have a lot of advantages. This study showed that both approaches are appropriate measures. 

As we could see that both forms for measuring JS can be seen as equivalent, they both help in deriving steps for interventions in organizations. In a next step of analyses, the sociological factors have to be regarded too. For example, if there are differences between groups like in gender [73] or in age [74], then the organization has to consider special actions and has to look up for the reasons. Here, the more detailed version of JS has advantages over the PAJS-FI. Typically, it can be advised in practice to think about the usage of the short versus long version of any scale in advance, if possible. If there are already some hypotheses regarding special facets, then the longer version could be helpful. In the other case when the facet-items had been used, then the next step in practice could be to investigate the results in small groups [75] (e.g., with so called “health circles”) where the effects are discussed to see which special aspects of the facets are important. 

Furthermore, the usage of approaches to measure JS can also be examined in other areas of work, which has been shown in other studies [76,77]. In practice, it is recommended to define at first the goals of measuring JS. In workplace health promotion projects or employee surveys, where efficiency is a main aim and a lot of other constructs are explored, a facet-item measurement is preferred. In cases where the facets of JS should be explored and differentiated, the usage of a facet scale measurement is advised. This study showed for practice that short and efficient measurements are appropriate to measure the facets of JS. 

## Figures and Tables

**Table 1:** Different forms of measurement of assessing job satisfaction (JS).

	Number of Items	
Form of JS	Multiple-Items	Single-Items
JS Global	Multiple-items for one global scale of JS	Single-item for global score of JS
JS Facets	Multiple-items for every facet of JS (facet scales)	Single-items for every facet of JS (facet-items)

**Table 2:** Comparison of the Profile Analysis of Job Satisfaction (PAJS) and the PAJS-Facet-Item (PAJS-FI) ^1^.

Facet of Job Satisfaction	PAJS—Facet Scale Measurement	PAJS-FI—Facet-Item Measurement
Information and communication	Three single-items (Sample item: I am … with the information about activities in the company.)	I am … with information and communication (activities in company, treatment of my suggestions, information from the management, information about innovations).
Demanding work	Three single-items (Sample item: I am … with my work domain.)	I am … with how demanding my job is (work domain, responsibility).
Relationship to direct colleagues	Four single-items (Sample item: I am … with the support of my direct colleagues.)	I am … with the relationship to my direct colleagues (team spirit, work atmosphere, division of work, support).
Relationship to direct supervisor	Four single-items (Sample item: I am … with the support of my supervisor.)	I am … with the relationship to my direct supervisor (support, openness for problems, arrangement of cooperation between colleagues, praise, criticism).
Organization and management	Three single-items (Sample item: I am … with the image of the company.)	I am … with the organization and management (effort regarding employees, participation possibilities, image).
Chances of making career	Five single-items (Sample item: I am … with my chances of moving up in my company compared to my colleagues.)	I am … with the chances of moving up and making career (compared to my colleagues, to colleagues from similar companies, to friends, possibility of making my desired career, possibility of further education).
Working conditions	Three single-items (Sample item: I am … with my working tools and materials.)	I am … with the working conditions (working tools and materials, working environment, work applications, personal design freedom).
Decision range	Three single-items (Sample item: I am … with my participation possibilities concerning my work domain.)	I am … with the decision range (classification of work tasks, possibility of participation).
Working and vacation times	Four single-items (Sample item: I am … with the planning of my vacation times.)	I am … with working and vacation times (working hours, consideration of wishes in organizing working hours, vacation times, organization of breaks).
Compensations of the employer	Three single-items (Sample item: I am … with the payment compared to my colleagues.)	I am … with compensations of the employer (financial, social, job security).
General framework conditions	Three single-items (Sample item: I am … with the extended benefits offered to me.)	I am … with extended benefits (flexible working-time models, burnout-package, workplace health management).

^1^ The three points “…” refer to the rating-scale from “1, very satisfied” to “5, unsatisfied”. An example for the rating of a single-item of the facet “organization and management” with a rating of “very satisfied” is “I am very satisfied with the image of the company”.

**Table 3:** Correlations between Profile Analysis of Job Satisfaction (PAJS, facet scales) and PAJS-Facet-Item (PAJS-FI, facet-items, N = 788) ^1^.

	1	2	3	4	5	6	7	8	9	10	11	12	13	14	15	16	17	18	19	20	21
1. Communication (FI)																					
2. Demanding (FI)	0.34																				
3. Colleagues (FI)	0.21	0.24																			
4. Supervisor (FI)	0.35	0.31	0.44																		
5. Organization (FI)	0.49	0.39	0.40	0.70																	
6. Career (FI)	0.44	0.44	0.26	0.38	0.51																
7. Conditions (FI)	0.32	0.34	0.20	0.20	0.32	0.38															
8. Decision range (FI)	0.43	0.47	0.32	0.40	0.53	0.46	0.39														
9. Time aspects (FI)	0.27	0.37	0.25	0.22	0.24	0.34	0.41	0.36													
10. Compensations (FI)	0.32	0.33	0.20	0.27	0.37	0.49	0.30	0.38	0.32												
11. Framework (FI)	0.34	0.36	0.25	0.29	0.37	0.37	0.44	0.45	0.50	0.36											
12. Communication (FS)	**0.73**	0.36	0.26	0.39	0.53	0.50	0.37	0.49	0.31	0.41	0.40										
13. Demanding (FS)	0.34	**0.76**	0.28	0.30	0.37	0.47	0.34	0.49	0.39	0.41	0.40	0.40									
14. Colleagues (FS)	0.25	0.25	**0.80**	0.43	0.43	0.33	0.23	0.35	0.28	0.28	0.27	0.31	0.31								
15. Supervisor (FS)	0.36	0.33	0.36	**0.82**	0.67	0.39	0.23	0.43	0.26	0.30	0.29	0.43	0.37	0.41							
16. Organization (FS)	0.52	0.40	0.28	0.37	**0.54**	0.54	0.39	0.47	0.33	0.55	0.41	0.64	0.47	0.37	0.43						
17. Career (FS)	0.41	0.45	0.28	0.35	0.49	**0.76**	0.37	0.47	0.33	0.47	0.38	0.52	0.53	0.36	0.41	0.58					
18. Conditions (FS)	0.29	0.35	0.27	0.20	0.28	0.35	**0.68**	0.33	0.35	0.29	0.40	0.36	0.37	0.32	0.24	0.41	0.40				
19. Decision range (FS)	0.42	0.56	0.32	0.38	0.48	0.44	0.40	**0.64**	0.44	0.39	0.48	0.51	0.64	0.38	0.43	0.50	0.48	0.37			
20. Time aspects (FS)	0.29	0.40	0.31	0.33	0.38	0.38	0.40	0.45	**0.66**	0.38	0.52	0.36	0.46	0.37	0.38	0.41	0.42	0.40	0.57		
21. Compensations (FS)	0.31	0.31	0.18	0.23	0.32	0.48	0.25	0.31	0.27	**0.74**	0.29	0.37	0.37	0.26	0.28	0.48	0.46	0.31	0.31	0.35	
22. Framework (FS)	0.34	0.38	0.30	0.27	0.40	0.44	0.50	0.43	0.46	0.49	**0.50**	0.43	0.47	0.38	0.34	0.56	0.51	0.54	0.53	0.57	0.46

^1^ Correlations between the facet-items of the PAJS-FI and their corresponding facet scales of the PAJS are printed in boldface. Communication = information and communication, Demanding = demanding work, Colleagues = relationship to direct colleagues, Supervisor = relationship to direct supervisor, Organization = organization and management, Career = chances of making career, Conditions = working conditions, Decision range = decision range, Time aspects = working and vacation times, Compensations = compensations of the employer, Framework = general framework conditions. FI = facet-items, FS = facet scales. All correlations are significant at *p* < 0.01.

**Table 4:** Factor loadings in a confirmatory factor analysis for Profile Analysis of Job Satisfaction Facet-Item (PAJS-FI, facet-items, N = 788) ^1^.

PAJS-FI Facet-Items	Factor Loading
1. Communication	0.79
2. Demanding	0.79
3. Colleagues	0.85
4. Supervisor	0.85
5. Organization	0.64
6. Career	0.78
7. Conditions	0.70
8. Decision range	0.69
9. Time aspects	0.69
10. Compensations	0.77
11. Framework	0.64

^1^ Standardized factor loadings are only shown for the facet-items of the PAJS-FI. Items of PAJS are not displayed in the table due to lack of space but are all higher than 0.60. Communication = information and communication, Demanding = demanding work, Colleagues = relationship to direct colleagues, Supervisor = relationship to direct supervisor, Organization = organization and management, Career = chances of making career, Conditions = working conditions, Decision range = decision range, Time aspects = working and vacation times, Compensations = compensations of the employer, Framework = general framework conditions. All standardized factor loadings are significant at *p* < 0.01.

**Table 5:** Correlations between facet-items (Profile Analysis of Job Satisfaction Facet-Item, PAJS-FI; on the left) and facet scales (Profile Analysis of Job Satisfaction, PAJS; on the right) with the external criteria Identification with Company, Work Engagement, Stress, and Resources (N = 788).

External Criteria		Left: Facet-Item (PAJS-FI)|Right: Facet Scale (PAJS) ^1^										
		Communication	Demanding	Colleagues	Supervisor	Organization	Career	Conditions	Decision Range	Time Aspects	Compensations	Framework
Identi-fication with Company ^2^	Recommend	0.39|0.45	0.39|0.42	0.28|0.35	0.35|0.35	0.42|0.65 *	0.43|0.45	0.35|0.35	0.41|0.45	0.34|0.39	0.38|0.33	0.36|0.44
	Identify	0.28|0.35	0.36|0.41	0.23|0.27	0.26|0.30	0.32|0.52 *	0.39|0.42	0.30|0.33	0.29|0.37	0.27|0.32	0.36|0.33	0.27|0.38
	Reapply	0.38|0.44	0.44|0.47	0.24|0.28	0.34|0.39	0.40|0.59 *	0.43|0.47	0.33|0.33	0.41|0.47	0.32|0.40	0.34|0.29	0.36|0.40
	Proud	0.33|0.40	0.43|0.46	0.26|0.28	0.33|0.37	0.38|0.64 *	0.44|0.48	0.35|0.34	0.36|0.42	0.29|0.36	0.41|0.37	0.34|0.44
Work Engage-ment	Vigor	0.31|0.40 *	0.50|0.53	0.32|0.36	0.36|0.41	0.44|0.53	0.41|0.45	0.34|0.38	0.47|0.54	0.35|0.45 *	0.34|0.27	0.38|0.46
	Dedication	0.33|0.39	0.61|0.66	0.29|0.31	0.33|0.38	0.40|0.56 *	0.41|0.49 *	0.34|0.37	0.48|0.56	0.35|0.42	0.33|0.29	0.37|0.49 *
	Absorption	0.31|0.38	0.52|0.56	0.23|0.27	0.30|0.35	0.38|0.52 *	0.39|0.44	0.32|0.34	0.41|0.49	0.29|0.36	0.31|0.26	0.33|0.43
Stress	Soc. em. stress ^3^	−0.33|−0.37	−0.35|−0.38	−0.32|−0.35	−0.32|−0.35	−0.38|−0.37	−0.34|−0.32	−0.33|−0.30	−0.41|−0.50	−0.34|−0.39	−0.30|−0.21 *	−0.34|−0.33
	Loss mean. ^4^	−0.38|−0.45	−0.48|−0.51	−0.36|−0.42	−0.45|−0.50	−0.50|−0.49	−0.43|−0.43	−0.37|−0.35	−0.49|−0.60 *	−0.38|−0.43	−0.37|−0.27 *	−0.41|−0.39
Resources	Ov. recovery ^5^	0.35|0.37	0.42|0.44	0.31|0.36	0.36|0.38	0.42|0.37	0.40|0.38	0.34|0.32	0.43|0.50	0.33|0.40	0.28|0.19 *	0.35|0.36
	Leisure/breaks	0.29|0.29	0.31|0.31	0.26|0.34 *	0.26|0.29	0.36|0.31	0.31|0.29	0.33|0.29	0.35|0.42	0.34|0.42	0.27|0.22	0.35|0.34
	Psychosoc. res. ^6^	0.22|0.26	0.20|0.24	0.68|0.72	0.42|0.39	0.39|0.29	0.30|0.31	0.22|0.23	0.34|0.32	0.22|0.27	0.22|0.18	0.25|0.31
	Work-rel. res. ^7^	0.39|0.45	0.49|0.55	0.39|0.40	0.49|0.48	0.50|0.43	0.41|0.46	0.37|0.35	0.59|0.63	0.34|0.42	0.29|0.23	0.38|0.43

^1^ Communication = information and communication, Demanding = demanding work, Colleagues = relationship to direct colleagues, Supervisor = relationship to direct supervisor, Organization = organization and management, Career = chances of making career, Conditions = working conditions, Decision range = decision range, Time aspects = working and vacation times, Compensations = compensations of the employer, Framework = general framework conditions. ^2^ Identification with Company: Single-item measures, Recommend = I would recommend the company as employer, Identify = I identify myself strongly with the company, Reapply = I would reapply at the company, Proud = I am proud to work at the company. ^3^ Soc. em. stress = social emotional stress. ^4^ Loss mean = loss of meaning. ^5^ Ov. recovery = overall recovery. ^6^ Psychosoc. res. = psychosocial resources. ^7^ Work-rel. res. = work-related resources. A star (*) denotes a significant differences between correlations of PAJS-FI and PAJS with external criteria.
